# Italian survey on the use of a multidisciplinary approach for age assessment of UAM: Alessandra Diodati

**DOI:** 10.1093/eurpub/ckac129.463

**Published:** 2022-10-25

**Authors:** A Diodati, F Curtale, C Mirisola, G Costanzo

**Affiliations:** INMP, Rome, Italy

## Abstract

**Issue:**

Many methods are used to assess the age of unaccompanied foreign minors (UAM). In Italy, in the frame of a new legislative asset, a multidisciplinary protocol has been adopted in July 2020 with the aim of ensuring that all UAMs are assessed uniformly throughout the country when their age is in doubt. The assessment is based on a multidisciplinary - social, psychological and physical - evaluation performed by a team of specialists. The local health authorities (ASL) carry out the assessment when requested by the Juvenile Court.

**Description:**

One year after the establishment of the protocol, INMP launched a national survey, through an online questionnaire, to investigate the formal adoption of the protocol by the ASL and their adherence in practice. The survey started on 10 January 2022 and closed on 8 March 2022.

**Results:**

Out of 118 ASL that were asked to participate, 102 (85%) answered. 37 declared to have a multidisciplinary team for age assessment. Of them, 18 use the formal protocol, 11 use an approach “in line” with the protocol and 8 have a forensic team, out of the rules of the protocol. Of the 65 that did not set up the team, 22 declared that they were ready to do so. 846 age assessment requests were reported and 687 were carried out. 398 migrants were recognized as minors, 222 migrants were not recognized as minors, and the age of 67 migrants remained uncertain.

**Lessons:**

The pandemic period prevented the effective adoption of the protocol by the ASL, that were strongly engaged in the COVID 19 response. There is still great variability in the way the age assessment of UAMs is conducted and in order to counteract the use of inadequate/outdated practices, it is necessary to actively promote and support the adoption of the protocol. In addition, a continuous comparison/dialogue between the multidisciplinary teams, the Juvenile Court, and the Police Headquarters is also needed, so that the procedure is requested only when necessary and carried out properly.

**Key messages:**

• In case of doubt, the age of all UAM has to be assessed in a uniform manner throughout the country.

• The adoption of the multidisciplinary protocol has to be actively promoted and supported to counter the use of inadequate or outdated assessment practices.

